# The Role of the Insula in Chronic Pain and Associated Structural Changes: An Integrative Review

**DOI:** 10.7759/cureus.58511

**Published:** 2024-04-18

**Authors:** Billy McBenedict, Dulci Petrus, Mariana P Pires, Anna Pogodina, Divine Besong Arrey Agbor, Yusuf A Ahmed, Jose Ittay Castro Ceron, Aishwariya Balaji, Ana Abrahão, Bruno Lima Pessôa

**Affiliations:** 1 Neurosurgery, Fluminense Federal University, Niterói, BRA; 2 Family Health, Directorate of Special Programs, Ministry of Health and Social Services, Windhoek, NAM; 3 Medicine and Surgery, University of Buckingham, Buckingham, GBR; 4 Internal Medicine, Richmond University Medical Center, Staten Island, USA; 5 Faculty of Medicine, Mansoura University, Mansoura, EGY; 6 Academic Medicine, Institute of Health Sciences, Autonomous University of the State of Hidalgo, Pachuca, MEX; 7 General Practice, Government Kilpauk Medical College and Hospital, Chennai, IND; 8 Public Health, Fluminense Federal University, Niterói, BRA

**Keywords:** functional magnetic resonance imaging, pain matrix, anterior cingulate cortex, chronic pain, insula

## Abstract

Chronic pain affects a substantial portion of the global population, significantly impacting quality of life and well-being. This condition involves complex mechanisms, including dysfunction of the autonomic nervous system, which plays a crucial role in pain perception. The insula, a key brain region involved in pain processing, plays a critical role in pain perception and modulation. Lesions in the insula can result in pain asymbolia, where pain perception remains intact but emotional responses are inappropriate. The insula is anatomically and functionally divided into anterior and posterior regions, with the posterior insula processing nociceptive input based on intensity and location before relaying it to the anterior insula for emotional mediation. Understanding the insula's intricate role in pain processing is crucial, as it is involved in encoding prediction errors and mediating emotional dimensions of pain perception. The focus of this review was on synthesizing existing literature on the role of the insula in chronic pain and associated structural changes. The goal was to integrate findings from various sources to provide a comprehensive overview of the topic. The search strategy included a combination of Medical Subject Headings (MeSH) and relevant keywords related to insula and chronic pain. The following databases were surveyed: PubMed, Embase, Scopus, and Web of Science. We identified a total of 2515 articles, and after following the Preferred Reporting Items for Systematic Reviews and Meta-Analyses (PRISMA) guideline based on eligibility criteria, 46 articles were used to synthesize this review. Our study highlights the pivotal role of the insula in chronic pain processing and associated structural changes, integrating findings from diverse studies and neuroimaging investigations. Beyond mere pain sensation, the insula contributes to emotional awareness, attention, and salience detection within the pain network. Various chronic pain conditions reveal alterations in insular activity and connectivity, accompanied by changes in gray matter volume and neurochemical profiles. Interventions targeting the insula show promise in alleviating chronic pain symptoms. However, further research is needed to understand underlying mechanisms, which can aid in developing more effective therapeutic interventions for pain.

## Introduction and background

Chronic pain afflicts over 1.5 billion individuals globally, significantly impacting their quality of life and overall well-being [1]. This persistent pain condition not only diminishes physical and cognitive function but also involves various mechanisms, including emerging evidence implicating dysfunction of the autonomic nervous system. In healthy individuals, nociceptive stimuli act as stressors, triggering the sympathetic nervous system's "fight or flight" response. This acute pain stimulation influences cardiovascular parameters such as heart rate, heart rate variability, and blood pressure by activating the sympathetic nervous system [2,3]. The increased sympathetic activity also elevates skin electrodermal activity, commonly measured as skin conductance [2]. However, in chronic pain states, the autonomic nervous system may become dysregulated, leading to attenuated autonomic responses to nociceptive or stressful stimuli. For instance, patients with irritable bowel syndrome exhibit significantly reduced heart rate variability compared to healthy individuals, indicating autonomic dysfunction [4]. Similarly, galvanic skin response reactivity tends to be lower in these patients [4]. Moreover, individuals with low back pain experience prolonged sympathetic response latency and decreased amplitudes compared to healthy controls [5]. Rheumatoid arthritis [6] and fibromyalgia [3] may also contribute to autonomic dysregulation.

**Figure 1:**
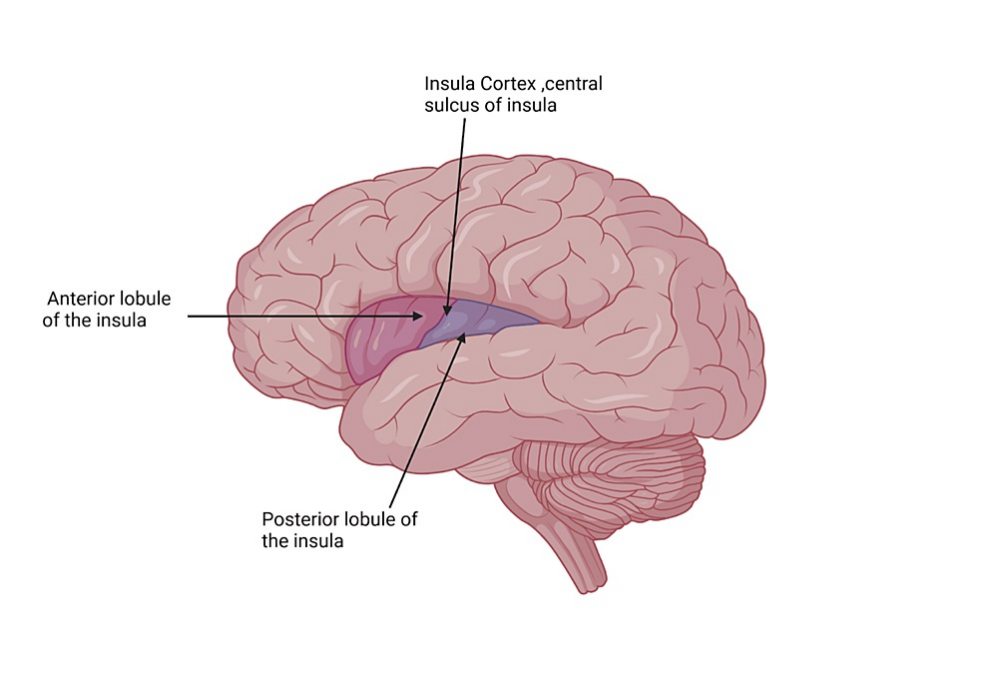
The anatomical location of the insula, including the cortex and both lobules of the insula Created with biorender.com

The insula (Figure 1) plays a crucial role in pain processing within the brain. It consistently participates in circuits associated with pain perception. Lesions in the insula can lead to pain asymbolia, a syndrome where pain perception remains intact but emotional responses to pain are inappropriate [7]. While the anterior cingulate cortex and insula show increased activity in response to painful stimuli, insular lesions suggest a more specific involvement in pain empathy. The insula's organization supports both affective and somatosensory functions, potentially suppressing pain perception by inhibiting the somatosensory cortex [7]. The insular cortex serves as a central hub connecting ascending, processing, and descending systems. It is anatomically divided into a "cognitive" (anterior) insula, receiving input from the anterior cingulate cortex, and a "sensorimotor" (posterior) insula, receiving input from somatosensory cortices. Nociceptive input is initially processed in the posterior insula based on pain intensity and location before being relayed to the anterior insula for emotional mediation [8].

The insula is intricately connected to modulatory brainstem centers through the tracts of the central sulcus, namely the insular cortex to locus coeruleus and insular cortex to subthalamic nucleus tracts. Since neither of these tracts exhibits connections to the thalamus, it is improbable that they serve as conduits for pain-related signals [8]. Instead, they are more likely to represent a secondary descending system, complementing the well-established dorsal pathway that is subject to regulation by pre- and orbito-frontal cortical regions. This aligns with the hypothesis of "top-down" modulation driven by the anterior insula under the control of the anterior cingulate cortex. While rodent models suggest an anterior origin for these connections, in humans, they originate from the posterior insula, implying a potential link between descending insula-driven pain modulation and objective pain intensity [8]. Experimental validation of this hypothesis is required.

Understanding the intricate role of the insula in pain processing is paramount due to its crucial significance within the brain's neural networks. As studies have demonstrated its involvement in encoding unsigned prediction errors (PEs), which provide insights into the magnitude and direction of unexpected outcomes, a need arises to delve deeper into its specific contribution to pain perception. Despite advancements in research, uncertainty persists regarding whether the insula's responses to PEs are exclusively tied to pain or extend to processing aversive events across various modalities. Moreover, the anterior insula (aINS) emerges as a pivotal hub for interoceptive awareness, particularly in pain regulation. Given its structural and functional connections with the limbic system and emotion-regulating regions, the aINS plays a central role in mediating the emotional dimensions of pain. Therefore, this comprehensive review was conducted to elucidate the role of the insula in chronic pain.

## Review

Materials and methods

The search strategy included a combination of Medical Subject Headings (MeSH) and relevant keywords related to ‘Insula’’ and ‘chronic pain.’ Boolean operators (AND, OR) were used to form search combinations. Eligible studies were those that investigated the role of the insula in chronic pain and associated structural changes. The systematic review utilized the Preferred Reporting Items for Systematic Reviews and Meta-Analyses (PRISMA) guidelines for reporting its findings.

Source information and search strategy

A literature search was performed on the following databases: PubMed, Embase, Scopus, and Web of Science. The search was conducted on the following dates: PubMed was accessed on January 14, 2024; Embase on January 14, 2024; and Scopus and Web of Science on January 15, 2024. The database coverage dates were from 2019 to 2024, and details regarding the search strategy can be found in Table 1. 

**Table 1:** Summary of the search strategy from the databases

Database	Search strategy	Filters used
PubMed	(Insula*[Title/Abstract]) AND (Pain[Title/Abstract] OR "Chronic Pain"[Title/Abstract])	Humans only, English language, exclude preprints, filter years 2019 - 2024
Embase	(insula:ab,ti OR insular:ab,ti) AND (pain:ab,ti OR 'chronic pain':ab,ti)	Humans only, English language, exclude preprints, filter years 2019 - 2024
Scopus	insula* Abstract: ( pain OR "chronic pain" )	Humans only, English language, exclude preprints, filter years 2019 - 2024
Web of Science	insula* (Abstract) AND (Pain OR "Chronic Pain") (Abstract)	Humans only, English language, exclude preprints, filter years 2019 - 2024

Inclusion and exclusion criteria

The inclusion criteria comprised studies involving humans of any age that provided insights into the role of the Insula in chronic pain, including associated structural changes. Accepted study designs included primary research studies, such as observational studies, clinical trials, experimental studies (including case studies), and reviews. Only peer-reviewed journal articles in English were considered for inclusion. Exclusion criteria encompassed non-human or animal studies, in vitro studies lacking direct relevance to the Insula and chronic pain, non-peer-reviewed articles, conference abstracts, editorials, and duplicate publications or studies reporting redundant data from the same research group.

Results

**Figure 2:**
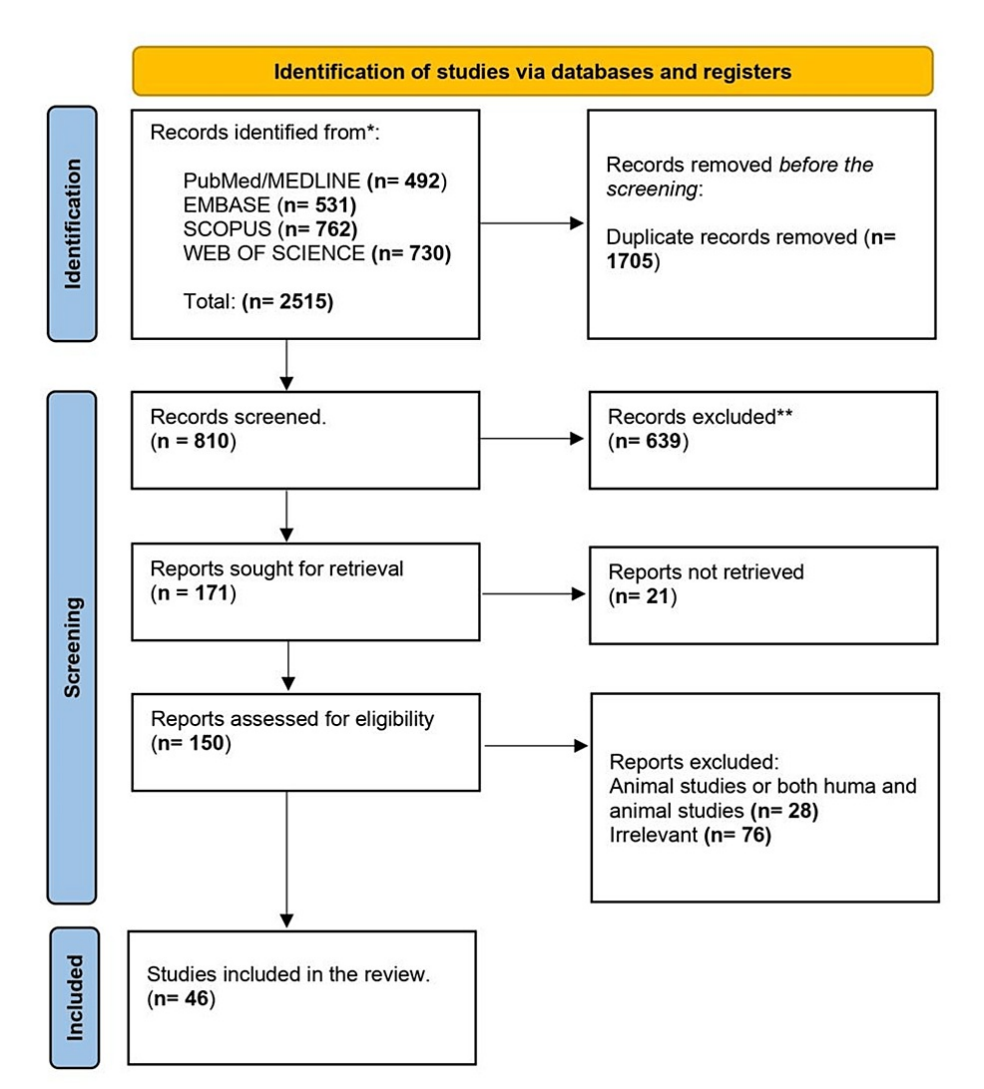
Preferred Reporting Items for Systematic Reviews and Meta-Analyses flow diagram indicating the steps taken to filter the articles for this review

We identified a total of 2515 articles (Figure 2), which included (1) 492 articles from PubMed; (2) 531 articles from Embase; (3) 762 articles from Scopus; and (4) 730 articles from Web of Science. The articles were imported into Zotero software, where duplicate papers were removed manually, leaving us with 810 records, and these were further reduced to 171 after screening the abstracts. Other texts such as conference abstracts were removed, leaving 150 articles, which were read in full. Based on eligibility criteria, 46 articles were used to synthesize this review. The focus of the review was on synthesizing existing literature on the role of the Insula in chronic pain and associated structural changes. The goal was to integrate findings from various sources to provide a comprehensive overview of the topic. The information extracted from the 46 articles was integrated into thematic analysis (Table 2) and presented as themes/topics in the discussion. Thus the critical appraisal of the search results was not performed.

**Table 2:** Studies that were used to synthesize this review, with their respective key results

Author	Type of Study	Results
Monroe et al. [9]	A cross-sectional study	Older females exhibit greater pain sensitivity at lower stimulus intensities than older males, accompanied by different patterns of brain activation and deactivation in regions associated with pain processing, particularly in the default mode network and areas associated with pain effect. Conversely, males exhibited greater activation than females in the primary (SI) and secondary somatosensory cortices (SII) and posterior insula.
Sanabria-Mazo et al. [10]	A parallel, pilot randomized controlled trial	Mindfulness + Amygdala and Insula Retraining demonstrated significantly greater reductions in functional impairment, anxiety, and depression, as well as higher improvements in mindfulness and self-compassion compared to relaxation therapy. There were also significant decreases in pain catastrophizing and psychological inflexibility and improvements in clinical severity and health-related quality of life at follow-up but not post-treatment.
Apkarian et al. [11]	A systematic review	The review explores brain regions associated with acute and chronic pain perception. It emphasizes distinctions between healthy individuals and those with chronic pain. Additionally, it delves into the impact of neurochemical factors on pain modulation. Notably, the anterior cingulate and insular cortices exhibit activity related to heat pain during Positron Emission Tomography or functional magnetic resonance imaging studies, which plays a role in emotional pain processing.
Mazzola et al. [12]	A prospective observational	Direct intracerebral stimulation of the SII area predominantly elicited somatosensory responses, with a similar rate of painful sensations compared to the insula but not the SI area, indicating distinct pain representations in SII and insular cortex.
Mazzola et al. [13]	A retrospective observational	Pain responses were rare (1.4%). They localized to the medial part of the parietal operculum and neighboring posterior insula, contrasting with findings from previous studies and challenging the notion of a distinct 'pain cortical area' described by Penfield.
Uddin et al. [14]	A literature review summarizing	The insular cortex, with its various subdivisions, serves diverse functions in humans, including sensory processing, affective responses, and high-level cognition, as evidenced by lesion case studies and neuroimaging research.
Sridharan et al. [15]	A cross-sectional	The study observed substantial activation in the Central Executive Network and concurrent deactivation of the Default Mode Network. Additionally, a third network involving the right fronto-insular cortex and anterior cingulate cortex became active when participants perceived significant auditory event boundaries.
Huang et al. [16]	Retrospective and cross-sectional	Menstrual migraine without aura patients exhibited decreased degree centrality in the right insula and altered directional connectivity between the insula and various brain regions associated with cognitive processes and emotional perception, suggesting disruption in insula resting-state functional networks in Menstrual migraine without aura.
Sandström et al. [17]	Cross-sectional comparative	During painful joint stimulation, Rheumatoid arthritis patients showed significantly less activation in brain regions associated with pain and somatosensory processing, including the anterior insula, compared to healthy individuals. Within the Rheumatoid arthritis patient group, brain activation in response to painful stimulation was reduced over the disease-affected joint compared to the non-affected thumbnail, affecting bilateral S1, bilateral S2, and the anterior insula.
Pujol et al. [18]	Cross-sectional comparative	Osteoarthritis patients exhibited weaker local connectivity in the insula, while fibromyalgia patients showed weaker connectivity in the sensorimotor cortex, indicating different patterns of cortical functional alterations in these conditions.
Tang et al. [19]	A comparative meta-analysis	In major depressive disorder adult patients, there was increased resting-state functional connectivity between the amygdala, the right hippocampus/parahippocampus, and the right inferior temporal gyrus. However, decreased resting-state functional connectivity was observed between the amygdala and the bilateral insula in major depressive disorder adult patients compared to major depressive disorder adolescent patients.
Nan et al. [20]	Cross-sectional study	Central alterations around the postcentral cortex in inflammatory bowel syndrome patients were associated with the level of visceral pain. They exhibited a better discriminative power than those around the whole brain and the insula when classifying the inflammatory bowel syndrome group and the healthy group.
Dongyang et al. [21]	A pilot double-blind, randomized cross-over study	Deep transcranial magnetic stimulation to the posterior superior insula resulted in significant pain relief, with 58.1% of patients responding to real deep transcranial magnetic stimulation compared to 19.4% responding to sham.
Mandloi et al. [22]	Clinical investigative study	Spinal cord injury participants had reduced functional connectivity of the anterior and middle insular gyri with several brain regions, such as the prefrontal cortex, the cingulate cortex, and the thalamus, compared to controls. Furthermore, The functional connectivity of the posterior insular gyrus with the cerebellum was positively correlated with pain intensity and interference in the spinal cord injury group.
Horing and Büchel [23]	Experimental study design	Participants (n=47) were investigated to understand the associations between conditioned stimuli and unconditioned stimuli related to painful heat or loud sound. They found activation in the anterior insula correlated with unsigned intensity prediction errors, signaling an unspecific aversive surprise regardless of modality. However, signed intensity prediction errors signals were modality-specific: dorsal posterior insula activation occurred following pain but not sound.
Kurokawa R et al. [24]	Case-Control study	The betweenness centrality in the Burning Mouth Syndrome group was notably higher in the left insula, right amygdala, and right lateral orbitofrontal cortex and conversely lower in the right inferotemporal cortex compared to healthy controls. However, no significant differences were observed between the two groups when examining other network measures, including clustering coefficient, node degree, and small-worldness.
Zhang et al. [25]	Case-Control study	A study examining functional connectivity of the posterior insula in patients with chronic daily pain observed increased connectivity between the posterior insula and the thalamus. However, there was a decreased connectivity between the posterior insula and the middle cingulate cortex. Further analyses within the pain network revealed that bilateral posterior insula showed heightened connectivity with bilateral thalamus, while left posterior insula exhibited reduced connectivity with the middle cingulate cortex. Additionally, the right posterior insula was negatively correlated with the left thalamus, while the left posterior cingulate cortex was positively correlated with the visual analog scale.
Teh et al. [26]	Observational, cohort study	Individuals with the irritable phenotype exhibited greater thalamus–insular cortex functional connectivity and reduced thalamus–somatosensory cortex functional connectivity compared to those with the non-irritable phenotype, suggesting distinct neural signatures for different pain phenotypes in diabetic polyneuropathy.
Strigo et al. [27]	Cross-Sectional study	Patterns in functional connection were identified in three subgroups, and they differed significantly in several psychological measures. The first subgroup was highly connected overall, characterized by functional connectivity from the nucleus accumbens, the anterior cingulate cortex, and the posterior cingulate cortex to the insula, and scored low on pain and trauma symptoms.
van Ettinger-Veenstra et al. [28]	Case-Control Study	Chronic Widespread Pain patients showed decreased connectivity in the inferior posterior cingulate cortex in the default mode network and increased connectivity in the left anterior insula/superior temporal gyrus in the salience network when compared to controls.
Wang et al. [29]	A Narrative Review	The insula is activated during pain and modulated by various factors, such as attention, expectation, emotion, empathy, and placebo. The insula is necessary for pain awareness and modulation, and pharmacological studies indicate that opioids, cannabinoids, and ketamine influence it.
Railton et al. [30]	Case-Control study	Functional connectivity between the secondary somatosensory cortex and left posterior insula was increased in hip osteoarthritis participants compared to controls; functional connectivity between bilateral posterior insula and motor cortices was significantly decreased in hip osteoarthritis participants. In response to painful hip activity, connectivity increased between the thalamus, periaqueductal grey matter, and brainstem.
Cerritelli et al. [31]	Randomized Controlled Trial	In the study, osteopathic manipulative treatment resulted in a distinct reduction in brain activity in areas related to interoception, including the bilateral insula, anterior cingulate cortex, left striatum, and right middle frontal gyrus, with a notable trend observed across three-time points.
Barroso et al. [32]	Cross-Sectional study	Patients with knee osteoarthritis exhibited heightened connectivity in primary sensory, motor, and parahippocampal regions, and the insula lost its centrality properties in knee osteoarthritis patients. At a subnetwork level, the modular identity of multiple brain regions was reconfigured in osteoarthritis. Notably, the middle frontal gyrus, insula, and cingulate cortex were affected.
Farrell et al. [33]	Integrative data-driven and hypothesis-testing approach	The study found ten significant negative genetic correlations between chronic pain and neuroimaging traits at the insula, posterior cingulate cortex, and pars triangularis. Among these, only one significant genetic causal proportion was found between mean insula thickness and chronic abdominal pain.
Ferraro et al. [34]	Comprehensive meta-analysis	The study revealed convergent neurofunctional dysregulations in chronic pain patients, particularly in the left anterior insula cortex. Dysregulated left anterior insular activity was considered a robust neurofunctional maladaptation and a potential treatment target in chronic pain disorders.
Becerra et al. [35]	Prospective cohort	In the study, the Default Mode Network showed a positive correlation with the Visual Analog Scale, suggesting that as pain scores decreased, the connectivity within the Default Mode Network also decreased. Specifically, this effect was observed in brain regions such as the orbital, precentral, superior medial frontal, cuneus, fusiform, temporal inferior, middle, anterior insula, and cerebellum.
Hemington et al. [36]	Prospective cohort	The results highlighted attenuated connectivity between the default mode network and salience network in chronic pain patients, particularly impacting the insula, a brain region crucial for processing pain and emotional regulation.
Cottam et al. [37]	Prospective Cohort	The findings underscore the complex alterations in brain connectivity in osteoarthritis patients, particularly involving the insula, contributing to pain processing and emotional regulation in this population.
Alves et al. [38]	A cross-sectional study	The study compared the conditions of the eyes open and eyes closed in patients with fibromyalgia. The findings revealed that fibromyalgia patients exhibited increased interhemispheric connectivity between insular areas. Specifically, there was heightened connectivity between the left insula and the right dorsolateral prefrontal cortex within the beta-3 frequency band.
Kaplan et al. [39]	Cohort	In fibromyalgia and rheumatoid arthritis patients, increased functional connectivity of the insula–left inferior parietal lobule, left inferior parietal lobule–dorsal anterior cingulate, and left inferior parietal lobule–medial prefrontal cortex regions correlated with higher levels of erythrocyte sedimentation rate (all family-wise error rate corrected P < 0.05).
Berman et al. [40]	Cohort	The study investigated brain activity in irritable bowel syndrome patients and healthy controls during rectal distention using functional magnetic resonance imaging. During anticipation of rectal distention, the insula showed decreased activity in healthy controls. In the irritable bowel syndrome patients, the insula showed less decrease in activity compared to controls during anticipation, suggesting a weaker calming response.
Zhu et al. [41]	Cohort	The study observed consistent activation in the insular cortex, particularly the anterior portion, which plays a pivotal role in integrating somatic and visceral information, as well as processing emotional salience.
Neeb et al. [42]	A double-blinded, randomized, placebo-controlled,	There was a significant increase in functional connectivity after active transcranial direct current stimulation within the visual medial and the right frontoparietal network being connected with the amygdala, the insula, and the primary somatosensory cortex, indicating central pain mechanisms in inflammatory bowel disease patients.
Tso et al. [43]	Cohort	Migraineurs showed increased connectivity between primary visual and auditory cortices and the right dorsal anterior insula, between the dorsal pons and the bilateral anterior insulae, and between the right and left ventral anterior insulae.
Chen et al. [44]	Systematic Review	The analysis identified decreased density in the orbitofrontal, frontal, insular, and parietal cortices. These findings suggest widespread structural alterations associated with Medication Overuse Headache pathology.
Coppola et al. [45]	Cohort	Interictally, migraine without aura patients showed significantly lower gray matter density in the right inferior parietal lobule, right temporal inferior gyrus, right superior temporal gyrus, and left temporal pole compared to healthy volunteers. Ictally, gray matter density increased in the left temporal pole, bilateral insula, and right lenticular nuclei, with no areas showing decreased gray matter density
Al Qawasmeh et al. [46]	Meta-analysis	Migraine without aura patients showed a grey matter volume reduction in the insula and anterior cingulate, whereas migraine with aura patients showed a grey matter volume reduction in the cerebellum, cingulate gyrus, and insula.
Wang et al. [47]	Cohort	Primary trigeminal neuralgia patients exhibited decreased trigeminal nerve volume compared to controls, alongside reduced gray matter volume in pain-associated regions such as the insula, secondary somatosensory cortex, hippocampus, dorsal anterior cingulate cortex, precuneus, and various temporal lobe areas.
Tanner et al. [48]	Prospective Cohort study	The study investigated the relationship between chronic pain intensity and brain structure in knee pain patients. They found no significant association between pain intensity and insula thickness, a brain region involved in pain processing. Interestingly, insula thickness was positively correlated with self-reported experiences of discrimination.
Denis [49]	Cross-sectional	Insular stimulation was observed to raise the heat pain threshold on both the ipsilateral (same side) and contralateral (opposite side) regions. Interestingly, stimulating the insular cortex not only triggered a pain response but also led to thermal pain inhibition. Although the effect size was modest, the difference was statistically significant.
Galhardoni et al. [50]	Double-blinded RCT	The study investigated deep repetitive transcranial magnetic stimulation for central neuropathic pain. While the main numeric rating scale score did not differ between groups, posterior superior insula deep transcranial magnetic stimulation showed promise. Compared to sham, posterior superior insula deep repetitive transcranial magnetic stimulation increased heat pain and warm detection thresholds, suggesting potential pain relief. Interestingly, posterior superior insula deep repetitive transcranial magnetic stimulation also caused a significant increase in paroxysmal pain symptoms, which needs further investigation.
De Groote et al. [51]	RCT	The study revealed an increased connectivity over time between the anterior insula region and the frontoparietal network. The increased functional connectivity between the left dorsolateral prefrontal cortex and the right anterior insula was significantly associated with the minimum clinically important difference value of the Pittsburgh Sleep Quality Index after three months of treatment.
Strath et al. [52]	RCT	The study found a connection between serum Vitamin D levels and insular volume and white matter surface area in middle-aged people experiencing knee pain. Those with optimal vitamin D levels (>30 ng/mL) exhibited greater gray matter volumes in the left inferior segment of the circular sulcus of the insula compared to individuals with insufficient (20–20.99 ng/mL) and deficient (<19.99 ng/mL) levels.
Zhao et al. [53]	Systematic review	The studies reviewed found changes in brain activity related to pain processing in chronic prostatitis/chronic pelvic pain syndrome patients. The right insula, which is involved in pain perception, showed functional activation in chronic prostatitis/chronic pelvic pain syndrome patients. Pain also impacted the connection between the insula and the motor cortex, suggesting altered pain processing.
Gussew et al. [54]	Cohort	Chronic low back pain patients showed potential changes in the insula. The morphological evaluation of anatomic brain data revealed a significantly decreased white matter volume of 17% and a non-significant trend for an increase in grey matter volume in the anterior insula of patients.

Discussion

Physiology of the Insula and Chronic Pain

Pain processing involves intricate neural networks spanning perception, cognition, and emotion [9]. This network consists of several brain structures jointly activated by painful stimuli. Among these structures, the amygdala is central in conditioning the learning processes associated with aversive and traumatic experiences. Furthermore, the insular cortex, along with other cortical areas such as the anterior cingulate and medial prefrontal cortex, contributes significantly to the higher-order control of amygdala-mediated fear conditioning [10]. The involvement of the insula in acute and chronic pain is well established, contributing to what is commonly termed the "pain matrix". This network comprises six primary regions - such as the primary and secondary somatosensory areas, insula, thalamus, anterior cingulate cortex, and prefrontal area - along with associated regions, including the primary and supplementary motor areas, posterior parietal area, posterior cingulate cortex, basal. ganglia, hypothalamus, amygdala, cerebellar peduncle nuclei, and periaqueductal gray matter [11].

Insular stimulation can elicit various sensory responses, including paraesthesia, non-nociceptive temperature sensations, and pain [12]. Pain induced by electrical stimulation is primarily associated with activation in the insular cortex and adjacent parietal operculum [13]. The insula maintains close connections with numerous cortical and subcortical regions, facilitating the coordination of diverse brain functions such as sensorimotor processing, pain perception, autonomic regulation, emotional awareness, attention, and salience. The insula possesses the capacity to prioritize critical information from a multitude of internal and external stimuli [14]. Its involvement extends to specific disease-related behaviors, such as those seen in migraine conditions. Functioning as a pivotal node within the salience network, the insula serves as a hub for switching between the salience, executive control, and default mode networks, potentially influenced by varying estrogen levels [15,16,55].

Literature reveals various aspects of the insula's involvement in pain processing: (1) Reduced activation in regions associated with pain and somatosensory processing of the anterior insula in rheumatoid arthritis [17]; (2) Weaker local connectivity in the insula due to decreased neural activity during metabolic recovery after repeated activation in osteoarthritis [18]; (3) Decreased amygdala functional connectivity with the insula in patients with primary dysmenorrhea [19]; (4) Changes around the postcentral cortex in irritable bowel syndrome patients, showing better discriminative power for visceral pain level than changes around the entire brain and insula [20]; (5) Lesions in the posterior insula reduce pain perception across large body areas, while direct electrical stimulation of this region can induce nociceptive sensations [21]; (6) Disruption of default mode networks and abnormal connections in the posterior insula in neuropathic pain [22]; (7) Decreased effective connectivity of the right precuneus, right inferior occipital gyrus, and left inferior parietal gyrus to the right insula in menstrual migraine without aura patients, along with an interrupted pathway from the frontal lobe to the insula, possibly associated with emotional regulation and inhibitory control of pain perception disorder [16,56,57].

The insula plays a crucial role in pain processing in the brain, with studies demonstrating its involvement in encoding the magnitude of unexpected outcomes, known as unsigned prediction errors [23]. Functionally connected with primary and secondary somatomotor cortices and the medial thalamus, the posterior insula processes touch, pain, and thermal stimulation and play a critical role in interoception, contributing to the subjective experience of pain [24]. The posterior insular area receives a substantial portion - more than 40% - of the thalamic fibers directed to the insula [58]. The insular cortex is a crucial interface for integrating multimodal information from internal and external sources, with extensive connections to cortical and subcortical brain areas, making it a core component of the pain network. Within the insula, the posterior insula is particularly important for pain perception and generation, functioning as part of the lateral pain matrix. The thalamus, acting as a relay for information exchange between cortical and subcortical regions, is pivotal in regulating consciousness. It receives projections from multiple ascending pain pathways and modulates nociceptive information, primarily involved in the sensory discriminative and affective motivational aspects of pain [25].

Regarding insula anterior cingulate cortex connectivity, enhanced connections between the limbic sensory cortex (insula) and the limbic motor cortex (cingulate) are associated with heightened affective experiences and greater motivation to modulate pain perception [26]. Furthermore, the anterior insula is a crucial center for interoceptive awareness, particularly pertinent to pain processing and regulation. Structurally and functionally linked to the limbic system and other areas involved in emotional regulation, the anterior insula plays a central role in the emotional dimensions of pain [24]. Research has demonstrated that weaker connections during experimental pain tasks correlate with greater subjective pain and avoidance responses in individuals with combat trauma and post-traumatic stress disorder [27]. In individuals with chronic widespread pain, increased pain sensitivity is associated with increased connectivity between the left and right anterior insular cortex and between the right anterior insular cortex and left lateral parietal cortex [28]. Nasal administration of oxytocin has been shown to increase insular activity in men but decrease it in women [29].

The insula, pain and structural-functional changes

**Figure 3:**
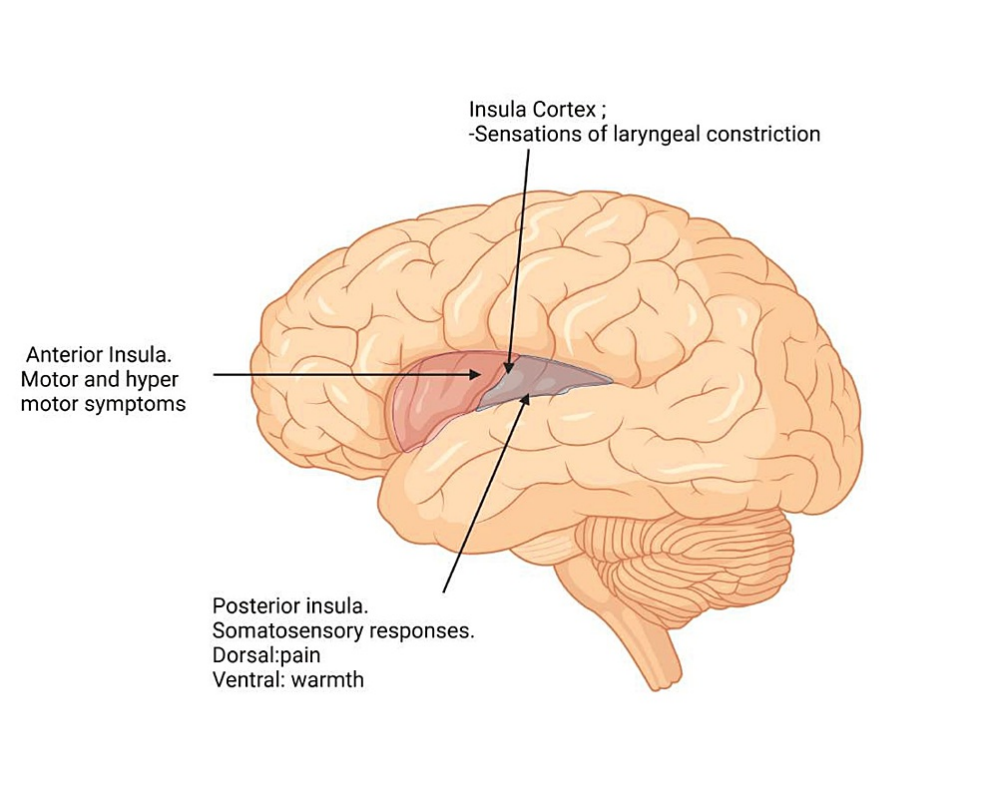
The anatomical position of insular lobes in relation to frontal, temporal, and parietal lobes of the brain and their respective functional significance Created with biorender.com

A study comparing the functional connectivity of pain-related brain regions before and after hip exercise using resting-state functional magnetic resonance imaging found significant differences in patients with hip osteoarthritis. Specifically, these patients exhibited reduced functional connectivity between the bilateral posterior insula and motor cortices, along with increased connectivity between the secondary somatosensory cortex and the left posterior insula compared to controls. Furthermore, painful hip activity led to increased connectivity between the thalamus, periaqueductal gray, and brainstem. Given the fundamental role of the insula in pain processing (Figure 3), these results are particularly intriguing. They suggest that patients with hip osteoarthritis have altered functional connections between brain regions associated with pain, and involvement in painful activities modulates some of these connections. The distinct lateralization of the left posterior insula and its associated functional connectivity patterns offer an objective means to assess pain perception and pain modulation strategies in patients with hip osteoarthritis [30].

Francesco Cerritelli and Piero Chiacchiaretta observed that knee osteoarthritis impacts brain function and structure, affecting areas involved in sensory, affective, and cognitive-evaluative discrimination. Gray matter abnormalities, including those in the lateral prefrontal cortex, parietal lobe, anterior cingulate cortex, insula, and limbic cortex, are prevalent in knee osteoarthritis patients [31]. Pain associated with osteoarthritis is associated with disruptions in both local and whole-brain functional connectivity and significant alterations in nodal connectivity have been observed in regions such as the insula, parahippocampal gyrus, and S1/M1 regions, suggesting a widespread functional reorganization throughout the brain [32]. This suggests that osteoarthritis may impair the brain's information exchange capacity. Pain intensity, a critical concern in osteoarthritis, correlates with changes in distributed nodal functional connectivity, a consistent finding across knee and hip osteoarthritis cases [32].

Recent studies have revealed enhanced functional connections linking the bilateral posterior insula and thalamus among individuals suffering from chronic discogenic pain, notably emphasizing the connectivity between the posterior insula and the thalamus. These connections have a positive correlation with the severity of the pain felt. Furthermore, chronic pain appears to coincide with reductions in insula volume and cortical thickness, precipitating changes in brain morphology. Collectively, these findings underscore a nuanced interplay between brain connectivity and morphology in chronic pain disorders, offering valuable insights into underlying mechanisms and pathways for targeted interventions [33].

The left anterior insula cortex detected consistent neurofunctional dysregulation in patients with chronic pain. Using a combination of seed-based resting-state functional connectivity analysis from a comprehensive public dataset and a task-based meta-analytic approach, we identify the anterior insula as a central node within a bilateral insula-fronto-cingulate network. extended that resembles the relevance network. Furthermore, meta-analytic decoding revealed a high probability of specific activation of this region during pain-related processes, although its involvement in autonomic processes cannot be ruled out. These results collectively suggest that dysregulated activity in the left anterior insula represents a robust neurofunctional maladaptation and a potential target for the treatment of chronic pain disorders [34]. Studies on chronic pain patients consistently show heightened connectivity between the right anterior insula, a key component of the salience network, and the central executive network [35,36,37]. The anterior insula is critical in the salience network, responsible for detecting and filtering important stimuli. Increased cerebral blood flow in the anterior insula correlates with higher pain intensity, and a shift towards increased salience network activity and decreased default mode network activity is observed during high-pain conditions. This suggests an interaction between mind wandering and ongoing pain through the interplay of default mode network and salience network.

Alves et al. explored the connectivity patterns in fibromyalgia patients under different conditions: eyes open and eyes closed. Fibromyalgia patients exhibited heightened connectivity between interhemispheric insular cortices, suggesting improved integration of sensory information and the sensory discriminative aspects of pain [38]. Additionally, when comparing eyes open and eyes closed conditions, within the beta-3 frequency band, there was increased connectivity between the left insula and right dorsolateral prefrontal cortex. These findings shed light on the neural dynamics associated with pain processing in fibromyalgia patients. Kaplan et al. found that fibromyalgia patients showed significant changes in critical brain hubs, particularly in regions linked to pain and sensory processing like the anterior insula, superior temporal gyrus, and primary motor cortex [39]. These regions acted as hubs exclusively in fibromyalgia patients, displaying heightened connectivity with other important nodes. Moreover, the anterior insula, mid, and anterior cingulate formed a higher-order hub structure called the rich club. The study also uncovered a neurochemical correlate of altered hub topology associated with pain perception, emphasizing the importance of these brain regions in information integration among fibromyalgia patients.

A study found distinct brain activation patterns in irritable bowel syndrome patients during painful distension compared to healthy controls [40]. Irritable bowel syndrome patients showed increased insula and ventrolateral prefrontal cortex (VLPFC) activation during stress, while dorsolateral prefrontal cortex and subgenual anterior cingulate cortex activation was reduced. Relaxation decreased activation in the right insula, cerebellum, putamen, and temporal regions during non-painful distensions and in the VLPFC, precuneus, and thalamus during painful distensions. After adjusting for anxiety levels, only cerebellum and precuneus activations remained significant. Patients with irritable bowel syndrome and functional constipation show heightened activity in the anterior insula during pain expectation, possibly due to inappropriate engagement of descending pain facilitatory mechanisms [40, 41]. Limited research on abdominal pain in inflammatory bowel disease patients using resting-state functional magnetic resonance imaging has shown reduced regional homogeneity in the insula among Crohn’s disease patients, suggesting potential neural correlates of chronic pain in inflammatory bowel disease. Furthermore, abnormal connectivity within the default mode network was observed in Crohn’s disease patients [42]. Increased connectivity is observed among the calcarine cortex, Heschl's gyrus, and the right dorsal anterior insula in individuals with migraine during interictal functional imaging. Specifically, heightened connectivity is noted between the right dorsal anterior insula and the left ventral medial part, as well as with regions of the left temporal lobe and the amygdala [43].

The use of medication to treat chronic pain can lead to structural changes in the insula. Chen et al. reported gray matter increases in the periaqueductal gray and the trigeminal afferent area in the midbrain, thalamus, and cerebellum, while a decrease in gray matter was noted in the insula in medication overuse for headache [44]. Research has indicated that migraine alters the activity of brain structures involved in memory processing and consolidation, including the insula [43,45]. Al Qawasmeh et al. found that patients with migraine without aura exhibited a reduction in grey matter volume in the insula and anterior cingulate, while those with migraine with aura showed grey matter volume reduction in the cerebellum, cingulate gyrus, and insula [46]. Insular changes in chronic pain, particularly in patients with trigeminal neuralgia, involve modifications in gray matter volume. Trigeminal neuralgia patients, especially those with longer pain duration, exhibit reduced grey matter volume in regions such as the insula, hippocampus, caudate, and temporal cortex. This suggests ongoing neuronal loss, particularly in the insula, impacting cognitive abilities and emotional control. Changes in gray matter volume related to primary trigeminal neuralgia have been linked to alterations in the volume of the left insula, resulting in increased pain scores [47].

A 2022 voxel-based meta-analysis revealed consistent bilateral insular gray matter volume reduction post-SCI. Stronger functional connectivity between the insula and thalamic sub-regions correlated with higher neuropathic pain intensities, suggesting that changes in gray matter volume post-SCI may primarily result from neuropathic pain rather than the initial spinal cord trauma [22]. A study revealed that a continuous measure of pain severity, Characteristic Pain Intensity, displayed an inverse correlation with cortical thickness in several brain regions, including the frontal, insular, and somatosensory areas. Furthermore, individuals classified with a high chronic pain stage exhibited thinner cortices throughout the entire brain, including the dorsolateral prefrontal cortex, when compared to their peers with a low chronic pain stage [48].

Insular stimulation and pain processing

The degree of pain relief following motor cortex stimulation is associated with the availability of opioid receptors within the human insula. Essentially, the insula exhibits a pro-nociceptive function when hyperactive, as seen in instances of acute and chronic pain. Interventions that suppress activity in this brain region, such as local lesions or electrical stimulation, induce antinociceptive effects by reducing its activity [21]. Insular stimulation has also been shown to induce thermal pain inhibition, suggesting a potential increase in pain threshold. The involvement of the insula in neuropathic pain pathophysiology may stem from lesions affecting spinothalamic tract projections, resulting in alterations in pain-processing brain areas. Such lesions could lead to deafferentation of insular pain recipients, causing functional disinhibition and increased processing of ascending inputs via mesial pain pathways [49].

Galhardoni et al. investigated the analgesic effects of stimulation of the anterior cingulate cortex and the posterior superior insula, compared against sham deep repetitive transcranial magnetic stimulation in patients with central neuropathic pain after stroke or spinal cord injury [50]. The posterior superior insula deep repetitive transcranial magnetic stimulation group exhibited a significant increase in heat pain threshold post-treatment, while the anterior cingulate cortex deep repetitive transcranial magnetic stimulation group showed a significant decrease in anxiety scores. Intraoperative stimulation of the human insula during stereo-EEG procedures has been shown to induce antinociceptive effects, while inhibition of the insula in experimental studies has led to antinociceptive responses [50]. One potential intervention for chronic pain is transcranial direct current stimulation targeting different cortical regions. Transcranial direct current stimulation delivers a low-intensity electrical current via scalp electrodes, modulating activity in underlying brain regions. Previous studies hypothesized that transcranial direct current stimulation over the primary motor cortex (M1) could reduce pain by influencing connections with regions such as the thalamus, brainstem, cingulate gyrus, prefrontal cortex, and insula. However, the exact mechanisms are not fully understood.

Despite the strengthening connectivity observed during high-frequency spinal cord stimulation, the relationship between the right anterior insula and the posterior cerebellum remains unclear despite the recognized active role of the cerebellum in acute and chronic pain [59]. Studies utilizing maneuver models to induce low back pain have limitations, potentially introducing order effects and lacking functional magnetic resonance imaging data from healthy controls. Nonetheless, the altered amplitude of low-frequency fluctuations in chronic low back pain patients with high pain indicates a link to differences in back pain intensity, though the physiological significance of altered amplitude of low-frequency fluctuations warrants further investigation. Posterior insula stimulation holds promise for use in patients with chronic pain. Existing evidence primarily stems from studies involving epileptic patients who underwent stimulation before epilepsy surgery. The posterior insula, due to its unique anatomical and functional properties, close connections with the thalamus, and associations with limbic and multisensory cortices, merits recognition as a third somatosensory cortex that contributes to the overall perceptual experience related to pain [60]. De Groote et al. employed resting-state functional magnetic resonance imaging in patients receiving regular treatment for back and/or leg pain [51]. Their study revealed heightened connectivity over time between the anterior insula, and regions within the frontoparietal network and central executive network. Following three months of high-frequency spinal cord stimulation, improvements in the Pittsburgh Sleep Quality Index's minimum clinically important difference value were associated with enhanced functional connectivity between the left dorsolateral prefrontal cortex and the right anterior insula.

Neuroimaging of the insula in neuropathic pain

Neuroimaging studies have consistently shown insular activation across various noxious modalities and body parts, indicating its role as a central hub for processing pain stimuli regardless of the source [52]. Functional magnetic resonance imaging offers a non-invasive means of measuring neuronal activity in the human brain by detecting changes in activity and generating signals. This technology has played a significant role in reshaping our understanding of chronic pain as a degenerative disease [22]. The findings of Zhao et al. highlight the advancements in neuroimaging techniques that have shed light on brain alterations in individuals with chronic pain disorders such as chronic prostatitis/chronic pelvic pain syndrome and chronic low back pain [53]. Recent investigations into chronic prostatitis/chronic pelvic pain syndrome mechanisms have centered on elucidating crucial pathological features. Findings from functional magnetic resonance imaging studies indicate heightened anterior insula activity in chronic prostatitis/chronic pelvic pain syndrome patients, which correlates with the intensity of clinical pain.

Studies using functional magnetic resonance imaging have investigated spinal manipulation's impact on brain pain matrix regions, such as the insula, thalamus, and anterior cingulate cortex. These studies have shown increased functional activity in these regions following spinal manipulation. Gussew et al. researched pain matrix areas in low back pain patients, revealing that reduced levels of glutamate and glutamine indicate impaired glutamatergic neurotransmission associated with prolonged pain perception. Additionally, decreased levels of N-acetyl-aspartate and myo-inositol reflect impaired function in glial cells and neurons [54]. Our study has some limitations that warrant acknowledgment: (1) heterogeneity of chronic pain conditions: Chronic pain encompasses various etiologies and manifestations, potentially introducing heterogeneity that may not be fully addressed in the review; (2) potential bias in study selection even though the search was done systematically in the initial phases of the study; (3) the review process may have introduced selection bias, as it relies on published literature, possibly overlooking unpublished studies or those in languages other than English; (4) scope of included studies: the review may not encompass all relevant studies on the topic, potentially omitting important findings or perspectives.

## Conclusions

The discussion underscores the intricate role of the insula in chronic pain processing and associated structural changes, as explored in various studies and neuroimaging investigations. Pain processing involves a complex network of brain structures, with the insula playing a pivotal role in integrating sensory, emotional, and cognitive aspects of pain perception. The involvement of the insula extends beyond mere pain sensation to encompass functions such as emotional awareness, attention, and salience detection. Studies have revealed alterations in insular activity and connectivity in various chronic pain conditions. These alterations are associated with changes in gray matter volume, functional connectivity patterns, and neurochemical profiles, suggesting a multifaceted interplay between brain structure, function, and pain perception. Moreover, interventions targeting the insula, such as transcranial magnetic stimulation and spinal cord stimulation, have shown promise in alleviating chronic pain symptoms by modulating insular activity. However, further research is needed to elucidate the underlying mechanisms and optimize treatment approaches.

Despite the advancements in neuroimaging techniques and our understanding of insular involvement in chronic pain, there are limitations to consider, including the heterogeneity of chronic pain conditions, potential biases in study selection, and the scope of included studies. Future research should address these limitations and explore new avenues for investigating the role of the insula in chronic pain, with the aim of developing more effective therapeutic interventions for individuals suffering from chronic pain disorders.
